# Effect of thermal stability on wind turbine wakes: an experimental and analytical study

**DOI:** 10.1007/s10546-026-00972-x

**Published:** 2026-04-06

**Authors:** Arslan Salim Dar, Konstantinos Kotsarinis, Fernando Porté-Agel

**Affiliations:** 1 Wind Engineering and Renewable Energy Laboratory (WIRE), École Polytechnique Fédérale de Lausanne (EPFL), 1015 Lausanne, Switzerland; 2 Department of Marine Technology, Norwegian University of Science and Technology, Trondheim, 7052 Norway; 3 Aeronautics and Astronautics, Stanford University, 496 Lomita Mall, Stanford, CA 94305 USA

**Keywords:** Atmospheric boundary layer, Thermal stability, Wakes, Wind energy, Wind turbines

## Abstract

We present first results from the thermally stratified boundary layer wind tunnel at EPFL, Switzerland. Stable, neutral, and convective thermal stability conditions are developed in the wind tunnel using temperature control of the floor and the air at the inlet. Two turbines in fully aligned conditions are then used to study the effect of thermal stability on the wake flow. The effect of stability on the recovery of the single and cumulative wake is shown, with a faster (slower) recovery in the convective (stable) conditions compared to the neutral ones. Two-point spatial correlations are performed to characterize the effect of thermal stability on turbulent length scales in turbine wakes. The correlations are found to be stronger in the convective case, with a hint towards enhanced meandering in this case. The stable case shows smaller correlations compared to the neutral one, with a strong signature of tip vortices behind the first turbine. The experimental data is used to test the Gaussian analytical wake model. As the streamwise turbulence intensity is the same in all cases, the model is unable to differentiate between different stability conditions. To resolve this, the relations for wake growth rate, near wake length, and wake added turbulence are re-formulated in terms of the vertical turbulence intensity. The re-formulated analytical framework yields reasonable predictions of the single and cumulative wake velocity deficit for all stability conditions.

## Introduction

Wind farms are sited in the lowest part of the atmospheric boundary layer. Due to spatial constraints wind turbines within a farm experience wakes of the upstream ones. This results in power losses and higher fatigue loads for the in-wake turbines compared to the ones exposed to the undisturbed flow (Porté-Agel et al. 2020). Thermal stability is one of the key atmospheric characteristics that can affect the development of wind turbine wakes (Abkar and Porté-Agel 2015; Machefaux et al. 2016; Li et al. 2022). In terms of thermal stability, the atmospheric boundary layer can be divided into three categories: neutral, convective, and stable. The neutral boundary layer (NBL) is characterized by zero temperature gradients, and the turbulence is purely generated by the surface shear stress. The convective boundary layer (CBL) corresponds to situations where the surface of the earth is hotter than the air above it, and is characterized by enhanced turbulent mixing due to positive buoyancy effects. Finally, in the stable boundary layer (SBL), the surface of the earth is cooler than the surrounding atmosphere, leading to negative buoyancy effects and lesser turbulence.

Several studies have investigated the effect of thermal stability on the evolution of wind turbine wakes. Compared to a wind turbine wake in neutral conditions, the enhanced turbulence in the convective boundary layer leads to a faster recovery of the mean wake velocity deficit (Abkar and Porté-Agel 2015; Iungo and Porté-Agel 2014; Xie and Archer 2017; Zhang et al. 2013), whereas the reduced turbulence in the stable boundary layer tends to slow the recovery down (Aitken et al. 2014; Abkar and Porté-Agel 2015; Xie and Archer 2017; Hancock and Pascheke 2014b). While the effect of thermal stability is often coupled with turbulence intensity, Du et al. (2021) investigated the effect of stability for a certain hub height turbulence intensity. They showed that, for the same hub height turbulence intensity, the mean wake velocity deficit still shows a faster (slower) recovery in the convective (stable) conditions compared to the neutral one. They related this to the effect of thermal stability on both the lateral transport of turbulence and the flow scale of the turbulent structures.

The turbulence kinetic energy and meandering in the wake have also been shown to be affected by thermal stability. Xie and Archer (2017); Wu et al. (2023) showed that the magnitude and distribution of added turbulence kinetic energy in the turbine wake are strongly dependent on thermal stability. The peak in the added turbulence kinetic energy (TKE) appears closer to the turbine in convective conditions and moves further away from the turbine in neutral and stable conditions. In addition, under convective conditions, the added TKE is dispersed in both lateral and vertical directions, whereas under stable conditions, it is mostly confined around the upper wake edges with lateral stretching as the wake moves downstream. Wake meandering has also been shown to increase under convective conditions compared to the neutral and stable ones (Porté-Agel et al. 2020; Ning and Wan 2019). In this context, Keck et al. (2014) adopted the dynamic wake meandering model to account for different stability conditions.

Wind tunnels offer a unique capability of studying wind turbine wakes under controlled and reproducible flow conditions. They provide capabilities to understand the physics of the wake flow (Cal et al. 2010; Espana et al. 2012; Bastankhah and Porté-Agel 2017c), and the effects of different flow and turbine configurations on power production (Bastankhah and Porté-Agel 2019; Duan et al. 2023), and serve as benchmarks for numerical and analytical models (Krogstad and Eriksen 2013; Lin and Porté-Agel 2019). To generate thermal stability in wind tunnels, a specialized temperature control system is needed. This includes heating and cooling of the air at the inlet of the test section, combined with independent heating and cooling of the test section floor to generate temperature gradients in the flow (Ohya et al. 1996; Chamorro and Porté-Agel 2010; Hancock and Pascheke 2014a). This makes it challenging to simulate a thermally stratified boundary layer inside a wind tunnel. While a majority of the literature uses numerical simulations or field measurements to investigate wakes under stratification, a few wind tunnel studies on the topic have also been conducted (Chamorro and Porté-Agel 2010; Zhang et al. 2013; Hancock and Pascheke 2014b; Hancock and Farr 2014; Placidi et al. 2023). These studies have provided key insights into the effects of stability on the evolution of wind turbine wakes.

Analytical modeling of mean wake velocity deficit is popular in the wind energy community for wind farm layout optimization control. The Gaussian wake model (Bastankhah and Porté-Agel 2014, 2016) is one of the commonly used models in the wind energy community. This model, coupled with the linear wake superposition principle of Niayifar and Porté-Agel (2016), offers a fast and accurate approach to predict mean wake deficit in a farm. Abkar and Porté-Agel (2015) further adapted this model for different thermal stability conditions by accounting for the difference in the lateral and vertical wake expansion rates. In their study, however, the wake expansion rates were obtained from the simulation results. In this approach, thermal effects are implicitly accounted for by the change in turbulence intensity. Krutova et al. (2020) evaluated different analytical models (including the Gaussian model under discussion), and showed that proper tuning of model parameters was needed to get reasonable results under different stability conditions.

In this study, we perform a combined experimental and analytical investigation of the wakes of two fully aligned wind turbines under different thermal stability conditions. We present the first results from a newly commissioned thermally stratified boundary layer wind tunnel. The experimental results are used to provide useful insights into the physics of the wake flow and to demonstrate the capability of the wind tunnel to generate thermal stability conditions relevant to wind energy. The experimental data is used to assess the Gaussian wake model (Bastankhah and Porté-Agel 2014, 2016; Niayifar and Porté-Agel 2016) and improve the analytical framework for application to different thermal stability conditions. The rest of the article is organized as follows: sect. 2 provides details of the experimental facility, flow characterization, and wind turbine used in the study; sect. 3 presents results from the experiments, along with analytical modeling of the wake velocity deficit; finally, some concluding remarks from the study are given in sect. 4.

## Experimental Setup

The thermal control system is incorporated within the boundary layer wind tunnel at the WiRE laboratory at EPFL, Switzerland. The wind tunnel without any thermal effects has been used in numerous prior studies (Bastankhah and Porté-Agel 2016, 2017c; Dar and Gertler n.d.; Duan et al. 2023; Dar et al. 2025). The wind tunnel has a test section of dimensions 28 *m* $\times$ 2.6 *m* $\times$ 2 *m*, with an area contraction of 5:1 at the inlet. The wind tunnel is a closed-loop type, and the flow is driven by 130 *kW* fan. A series of meshes and honeycomb structures is installed before the test section to facilitate uniform flow with low turbulence (typically $\approx 1\%$) at the inlet. The wind tunnel features a newly commissioned temperature control system. The temperature control system comprises three elements: a dehumidifier to keep the air dry, a temperature control system for the air, and an independent temperature control system for the floor. In addition to these components, the wind tunnel is fully covered with an insulating foam material to prevent heat losses from the sides.

The air dehumidifier is installed at the exit of the wind tunnel test section. Approximately 10% of the air at the test section outlet goes through the dehumidifier in each loop. The heating or cooling of the air and floor starts once the absolute humidity drops below 5 *g*/*kg*. The test section floor is composed of a series of aluminum plates 40 *cm* long and covering the width of the test section. A heating and cooling system controls the temperature of each aluminum plate individually. For floor cooling, a solution of water and ethylene glycol (35% ethylene glycol) is circulated through the floor cooling circuit. For floor heating, electrical heaters are installed. Through this system, the minimum and maximum floor temperatures are 263*K* and 373*K*, respectively.

**Fig. 1:**
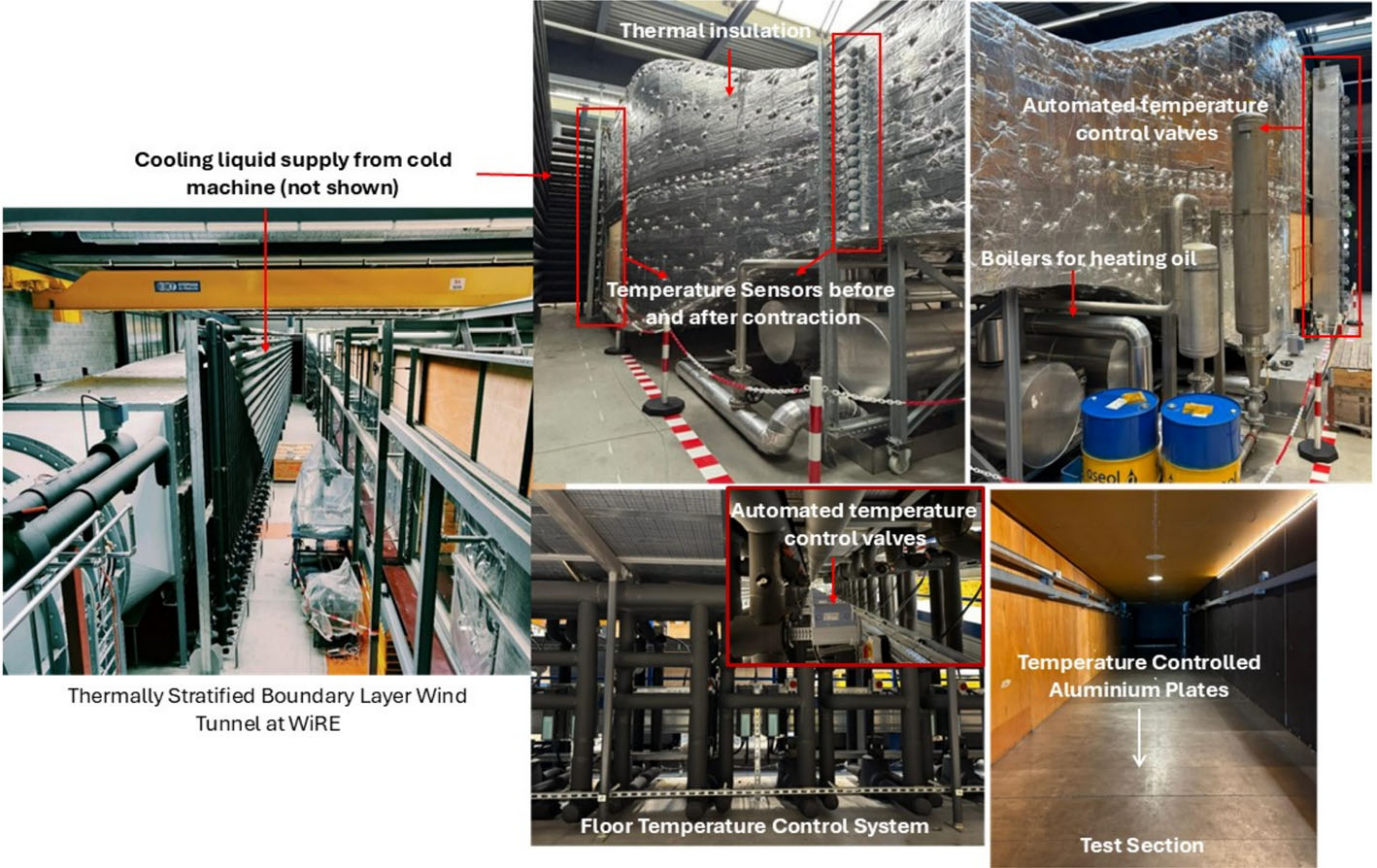
Snapshots of the wind tunnel and thermal control system with a description of key components.

**Fig. 2:**
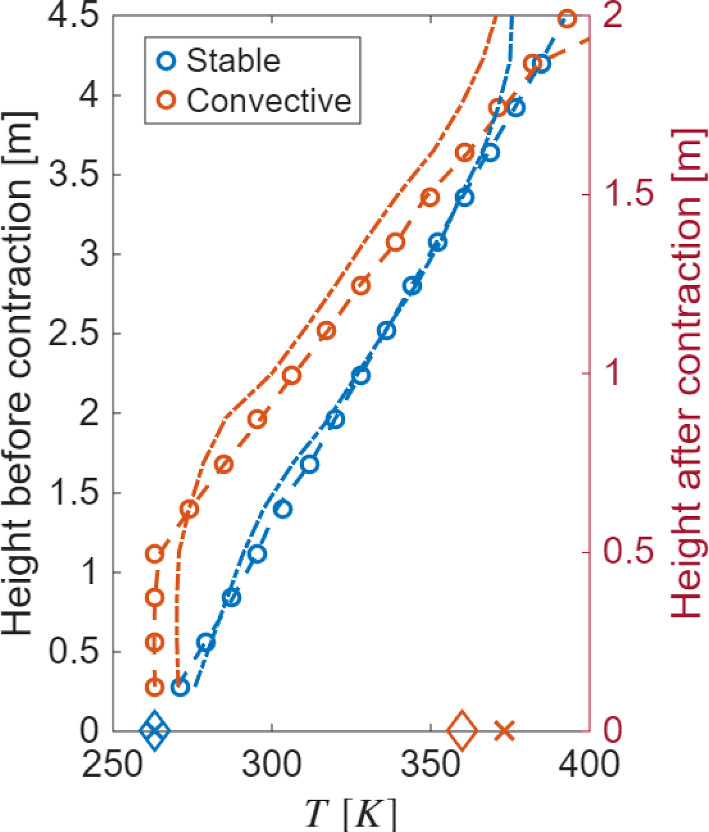
Temperature profiles for the air and test section floor. Target temperature profiles of the air before the wind tunnel contraction are marked by circles, and dashed lines show temperature before contraction; the height coordinate for circles and dashed lines is with respect to the bottom surface before contraction (left axis). Dashed-dotted lines show temperature after contraction, and the test section floor target temperature is marked by crosses (blue) stable and (orange) convective conditions, and diamonds for actual temperature. The height coordinate is with respect to the test section floor (right axis).

For heating and cooling of the air, heat exchangers are installed before the contraction of the wind tunnel. The temperature of the air is controlled at 16 equidistant levels along the height of the wind tunnel before the contraction. Two sets of heat exchangers are used: for air cooling, the heat exchangers use a water and ethylene glycol solution (same as the floor), whereas the heat exchangers for air heating use a specialized oil heated in boilers up to a temperature of 463 *K*. The minimum and maximum air temperatures are 263*K* and 393*K*, respectively. The operation of the heating and cooling circuits, and control of the temperatures in the air as well as the floor, is done through automatically controlled electronic valves. Two different temperature profiles for the air and test section floor are set up to develop stable and convective thermal stability. A picture of the wind tunnel together with a close-up view of key air and floor temperature control systems is shown in Fig. 1. In addition, Fig. 2 shows the design temperature profiles for the air at the start of the contraction and for the test section floor for different stability conditions. It also shows the actual air temperatures achieved by the thermal control system before and after the wind tunnel contraction, and at the floor. It can be seen that, before the contraction, the system is able to achieve target temperatures quite well. After the contraction, there is a slight change in the profile, especially for the convective case, however, the overall temperature gradient is well preserved. For floor, the actual and target temperatures are also very close. This verifies that the thermal control is capable of achieving the target temperature gradients in the flow. A third case with neutral stability is also investigated, for which no temperature control is performed.

Measurements are performed approximately 20 *m* downstream from the inlet of the test section, as it is the distance where the turbulent boundary layer is fully developed. Consistent with previous studies (Bastankhah and Porté-Agel 2016, 2017b; Dar and Porté-Agel 2022; Duan et al. 2023), a boundary layer is naturally developed due to the length of the test section without the use of any external tripping mechanism. Mean temperature profiles in the vertical direction are measured using a K-type thermocouple, with a wire diameter of 0.6 mm, in the middle of the span of the test section. At each measurement point, temperature measurements are performed for 60 *s* at a sampling rate of 1 *kHz*. Velocity measurements are performed using a two-dimensional two-component (2D2C) particle-image velocimetry (PIV) system. The PIV system used in the study is developed by LaVision and has been used in previous studies (e.g., see (Bastankhah and Porté-Agel 2016; Dar and Gertler n.d.; Dar et al. 2025)). Streamwise and vertical velocity components are measured to capture the boundary layer flow, and to measure the wake flow in a vertical (*xz*) plane passing through the center of the turbine rotor. The PIV system comprises an sCMOS camera (2560 $\times$ 2160 pixels) with a 50 mm lens, a 425 *mJ* Nd:YAG laser, and a programmable timing unit. Olive oil particles with a diameter on the order of micrometers are used as tracer particles. The images are acquired at a rate of 10 *Hz*, where a total of 1000 image pairs are used to obtain flow statistics. Image acquisition and processing are performed using DaVis software developed by LaVision. To remove any background noise, image pre-processing is performed using the ’subtract time filter’ operation in DaVis. Using this operation, we remove the minimum light intensity from images using a moving time window with a window length of 7 images. Image correlation is performed on reducing size windows with a window size of 64 $\times$ 64 pixels in the first window and 32 $\times$ 32 pixels in the final window. The correlation is obtained by two passes through each window size, and an overlap of 75% is kept between neighboring windows. To remove bad vectors, a post-processing using the universal outlier detection method (Westerweel and Scarano 2005) is performed on the velocity fields.

**Fig. 3:**
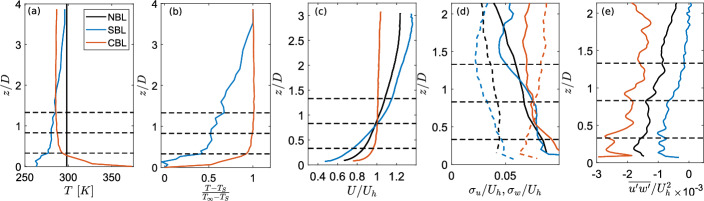
Boundary layer characteristics: (a) absolute mean temperature, (b) normalized mean temperature, (c) normalized mean streamwise velocity, (d) streamwise turbulence intensity (solid lines) and vertical turbulence intensity (dashed lines), and (e) normalized mean vertical momentum flux. Horizontal dashed lines show prospective rotor tips and hub locations.

Figure 3 shows the boundary layer characteristics for the three thermal stability conditions. The height coordinate is normalized by the turbine rotor diameter *D*, which is 15 cm in the current study. The mean absolute temperature shows a rapid decrease in temperature close to the surface, followed by a nearly uniform temperature profile in the convective case. The high temperature gradient close to the surface demonstrates the difference in the temperature of the surface and the air above it, whereas due to high turbulent mixing, the profile becomes uniform with the increase in the distance from the surface. For the stable case, the temperature increases almost linearly with the increase in the distance from the surface. The normalized temperature profile ($(T-T_S)/(T_\infty -T_S)$, with $T_S$ the mean surface temperature and $T_\infty$ is the mean temperature in the free stream, taken as the temperature at the top most measurement point) is also shown in Fig. 3 (b). This profile also shows the very high temperature gradient near the surface for the convective case, and the gradual increase in the temperature with height for the stable case.

The normalized mean streamwise velocity is also shown in Fig. 3 (c). All velocities are normalized by the mean streamwise velocity at the hub height $U_h$ of the turbine used in the study, which is 1.63 $ms^{-1}$, 1.37 $ms^{-1}$ and 1.32 $ms^{-1}$ for the neutral, convective, and stable cases, respectively. As expected, the convective case shows a reduction in the mean flow shear, whereas the stable case shows an increase in it compared to the neutral case. The streamwise and vertical turbulence intensities computed as $\sigma _u/U_h$ and $\sigma _w/U_h$, respectively, are also compared between different cases in Fig. 3 (d). Interestingly, the streamwise turbulence intensity is observed to be comparable between the three cases in the rotor projected area (marked by the horizontal dashed lines). The vertical turbulence intensity, on the other hand, showed a strong dependence on the thermal stability, with the highest values in the convective boundary layer. The vertical turbulence intensity decreases for the neutral and further for the stable boundary layers. Another thing to note is that in the neutral and stable boundary layers, the streamwise turbulence intensity is higher than the vertical one across the entire vertical extent of the measurements. For the convective boundary layer, however, the vertical turbulence intensity is greater than the streamwise one above a certain height from the surface. A similar observation was made in Abkar and Porté-Agel (2015). However, in their study, the crossover between the streamwise and vertical turbulence intensity happened at a larger height. This could be related to the strength of the stability. Finally, Fig. 3 (e) compares the normalized vertical momentum flux between different cases. As expected, the normalized mean vertical momentum flux is higher in the convective case and decreases for the neutral and stable cases.

In order to characterize the effect of thermal stability, several methods have been employed. As the velocity measurements are limited in the vertical direction, a bulk Richardson number $Ri_b$ is computed between the surface and the height furthest from the surface captured in the PIV measurements. The boundary layer height in neutral conditions is 0.38 *m* (height at which velocity reaches 99% of the free stream value). However, in other cases, estimating the height of the boundary layer may not be so straightforward and limited by the maximum height captured in PIV measurements. Therefore, following Howard et al. (2016), we define the bulk Richardson number $Ri_b = \frac{g \Delta z \Delta T_z}{\overline{T}U_z^2}$, where *g* is the gravitational acceleration, $\Delta z$ is the difference between the surface and the maximum height captured by the PIV, $\Delta T_z$ is the temperature difference between the surface and the maximum height of velocity measurements, $\overline{T}$ is the mean absolute temperature in the boundary layer and $U_z$ is the velocity at the height furthest from the surface captured in measurements. The resulting bulk Richardson number $Ri_b$ is approximately 0.15 in the stable boundary layer and -0.59 in the convective one. This indicates that we have weakly stable but strongly convective conditions in our experiments.

**Fig. 4:**
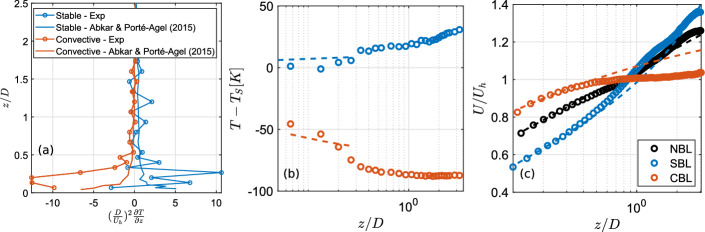
(a) Scaling between temperature gradient and turbine diameter to hub height velocity ratio, (b) Experimental (circles) and fitted (dashed lines) temperature difference from the surface, and (c) normalized mean streamwise velocity from experiments (circles) and fitted (dashed lines).

Following Hancock and Pascheke (2014a), the term $(D/U_h)^2\partial T/\partial z$ needs to be comparable between the full-scale and wind tunnel scale experiments to generate stability effects relevant for full-scale applications. Fig 4 (a) compares the above-described scaling term in the current experiments and the large eddy simulation of a real-scale turbine by Abkar and Porté-Agel (2015). As can be seen, the scaling shows higher values in the current experiments than in the simulation results of Abkar and Porté-Agel (2015). This indicates that the stability conditions in this study are relevant for real-scale wind energy applications.

Finally, we use the Monin-Obukhov similarity theory (MOST) to fit the velocity and temperature profiles and estimate the Obukhov length $L_0$. The fitting is done on the data within the lowest 10-15% of the boundary layer corresponding to the surface layer. According to MOST, the velocity profile is described as:

1 $$\begin{aligned} U = \frac{u_*}{\kappa }[ln(\frac{z}{z_0})-\psi _m(\frac{z}{L_0})], \end{aligned}$$

where

$$ \psi _m = {\left\{ \begin{array}{ll} -4.7 \frac{z}{L_0}, & SBL, \\ 0, & NBL, \\ 2ln[\frac{1}{2}(1+\xi )]+ln[\frac{1}{2}(1+\xi ^2)-2tan^{-1}(\xi )+\frac{\pi }{2}], & CBL, \end{array}\right. } $$

where $\xi = (1-15z/L_0)^\frac{1}{4}$. Similarly, the temperature profile is given as:

2 $$\begin{aligned} T-T_S = \frac{T_*}{\kappa }[ln(\frac{z}{z_{0,h}})-\psi _h(\frac{z}{L_0})], \end{aligned}$$

where

$$ \psi _h = {\left\{ \begin{array}{ll} -7.8 \frac{z}{L_0}, & SBL, \\ 0, & NBL, \\ 2ln[\frac{1}{2}(1+\xi ^2)], & CBL. \end{array}\right. } $$

The Obukhov length $L_0$ is given by:

3 $$\begin{aligned} L_0 = \frac{\overline{T}u_*^2}{\kappa g T_*}. \end{aligned}$$

In the above set of equations, $\kappa$ is the von Karman constant taken to be 0.4, $u_*$ is the friction velocity, $T_*$ is the temperature scale, $z_0$ is the aerodynamic roughness length, $z_{0,h}$ is the thermal roughness length, $z_{0,h}$ is the thermal roughness length, and $L_0$ is the surface Obukhov length. In order to obtain $u_*$, $T_*$, $L_0$, we used the iterative profile method described in Holtslag et al. (2014). Once these parameters have been obtained, they are input into equation 1 and 2 to obtain $z_0$ and $z_{0,h}$, respectively. The resulting fitted profiles are shown in Fig 4 by dashed lines. Table 1 lists all the parameters obtained for different boundary layers. As can be seen, the Obukhov length $L_0$ is smaller for the convective case than the SBL case, indicating stronger stability in the convective case than the stable one. This is consistent from the strength of thermal stability deduced from the bulk Richardson number.

**Table 1:** Key thermal stability parameters in the boundary layer

	$u_*$ ($ms^{-1}$)	$T_*$ (*K*)	$z_0$ (*m*) $\times 10^{-4}$	$z_{0,h} ($m) $\times 10^{-5}$	$L_0$ (*m*)	$Ri_b$
NBL	0.11	-	2.7	-	0	0
SBL	0.084	0.53	6.5	9	0.95	0.15
CBL	0.095	-4.8	1.3	7.4	-0.14	-0.59

A three-bladed horizontal axis wind turbine WiRE-01 (Bastankhah and Porté-Agel 2017a) is used in this study. The blade profile has a circular arc shape, with a 5% camber and 5% thickness with respect to the chord length. The turbine has a rotor diameter *D* of 15 *cm* and a hub height $z_h$ of 12.5 cm. The rotor is 3D printed, and is attached to a direct current machine (model: DCX10L), that serves as the generator. The power produced by the turbine is computed as: $P = (Q_{em} + Q_f)\Omega = (KI+Q_f)\Omega$, where $Q_{em}$ is the electromagnetic torque obtained by multiplying the generated current *I* by the torque constant *K* of the motor, $Q_f$ is the frictional torque obtained from Bastankhah and Porté-Agel (2017a), and $\Omega$ is the rotational speed of the rotor. The tip speed ratio $\lambda$ of the turbine is selected such that the power produced by the turbine is maximum. The tip speed ratio is adjusted individually for turbines in different cases and waked or un-waked situations. For wake measurements, two turbines are placed at a spacing of 8 rotor diameters in fully aligned conditions, and several fields of view are stitched together to capture the wake from the first turbine location to 8*D* downstream of the second turbine. As the PIV setup cannot be moved due to optical access restrictions in the wind tunnel, the turbine positions in the wind tunnel are changed to measure the wake of the two-turbine array. A similar approach has been used in previous studies (see, e.g. (Bastankhah and Porté-Agel 2016, 2017b)). The power coefficient $C_P = \frac{P}{0.5\rho AU_h^3}$ is computed for the turbine exposed to the three stability conditions. The resulting $C_P$ is 0.242, 0.23, and 0.218 for the neutral, convective, and stable conditions, respectively. It is to be noted that the power coefficient in these experiments is lower than what is usually measured for the WiRE-01 rotor (Bastankhah and Porté-Agel 2017a). This is due to the relatively lower Reynolds number ($Re_D = DU_h/\nu$) in the current study; $Re_D$ is 15400, 13100, and 13400 for the neutral, convective, and stable cases. The Reynolds number is kept low on purpose to enhance the thermal stability effects in the flow, a compromise often made for wind tunnel studies of stratified flows (Zhang et al. 2013; Hancock and Farr 2014).

## Results & Discussion

### Power Performance

**Fig. 5:**
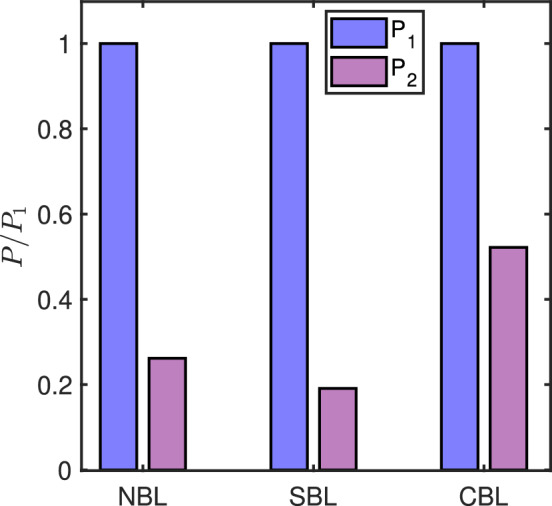
Mean power of the turbines normalized by the mean power of the upstream-most turbine for each thermal stability condition.

It is well known that wind turbines exposed to the wake of the upstream ones experience power losses due to the reduced available power (Porté-Agel et al. 2020). Here we characterize the effect of thermal stability on the power performance of an in-wake turbine. Fig. 5 compares the mean power of the two turbines in fully aligned conditions, normalized by the power of the upstream-most turbine in each stability condition. For neutral conditions, the power of the in-wake turbine drops to 26% compared to the upstream one. This is a relatively high drop in power considering the inter-turbine spacing of 8*D*, and can be related to the coupled effect of reduced available power and aerodynamic performance of the turbine at a lower Reynolds number in the wake. However, for the sake of comparison, the relative decrease or increase in the power performance in other thermal stability cases would still be interesting. For the stable conditions, we observe a further decrease in the power performance of the in-wake turbine, as it produces only 20% power compared to the upstream turbine. This is due to the fact that in stable conditions wake effects are stronger, which lead to lesser available power for the downstream turbine. Finally, in convective conditions, the in-wake turbine shows the highest efficiency, producing about 52% power compared to the upstream turbine. This can be related to the faster recovery of the wake in convective conditions.

**Fig. 6:**
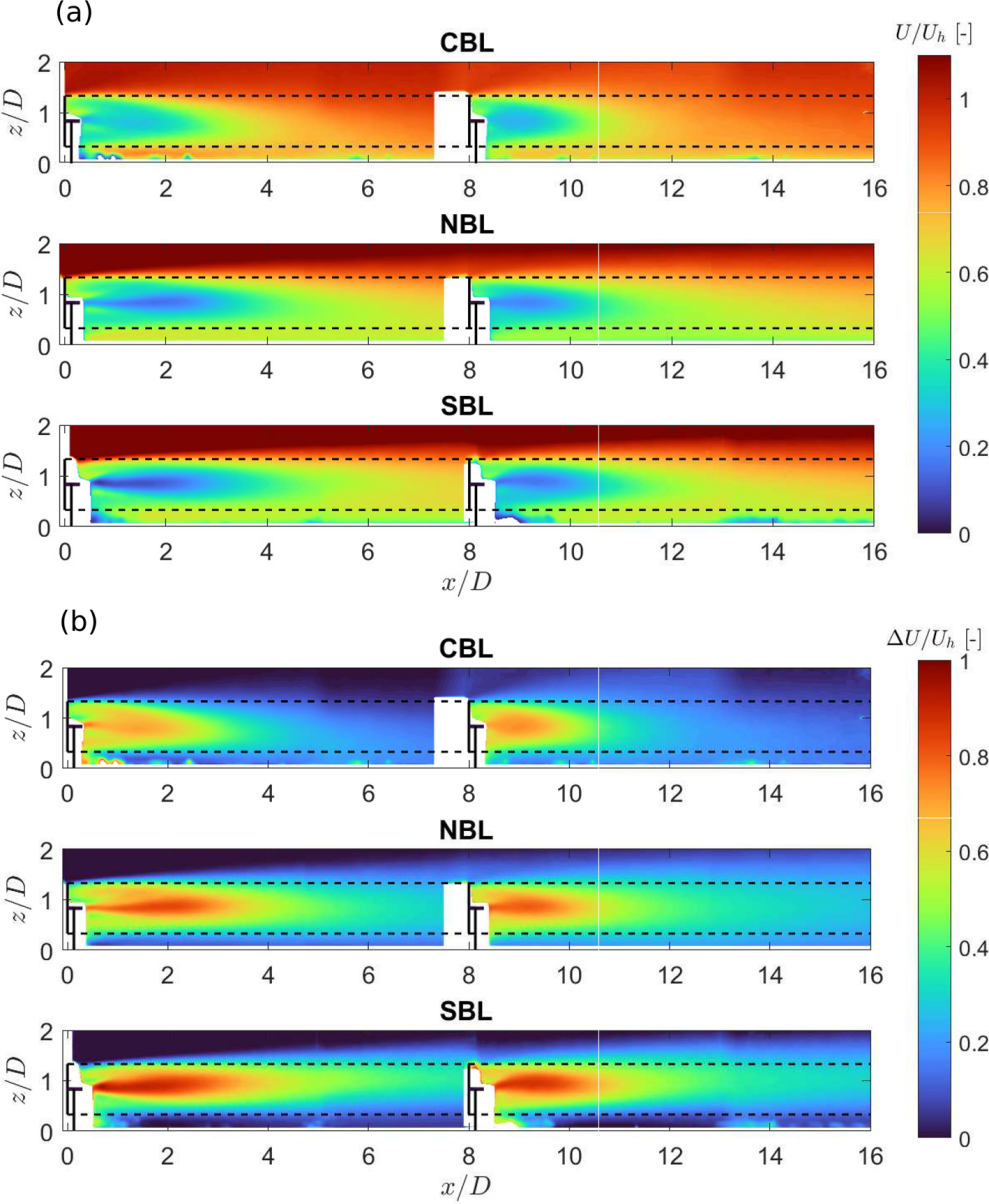
Contours of the normalized averaged streamwise velocity (a), and the normalized averaged streamwise velocity deficit (b) in the turbine wake for different thermal stability conditions.

### Time-averaged Wake Characteristics

Figure 6 (a) shows the contours of the normalized averaged streamwise velocity in the turbine wake under different thermal stability conditions developed in the wind tunnel. The CBL case is observed to show the highest normalized averaged streamwise velocity in the wake of the single turbine, as well as in the cumulative wake of two turbines. The SBL case, on the other hand, shows slightly lower normalized averaged streamwise velocity in the turbine wake compared to the NBL case. To further isolate the effect of the turbine on the flow, we compute the streamwise velocity deficit $\Delta U = U_{in}-U_w$, where $U_{in}$ is the streamwise velocity in the incoming boundary layer flow and $U_w$ is the streamwise velocity in the turbine wake. Figure 6 (b) shows the contours of the normalized averaged streamwise velocity deficit in the wake of the two fully aligned wind turbines. Consistent with previous studies (Zhang et al. 2013; Hancock and Pascheke 2014b; Abkar and Porté-Agel 2015; Machefaux et al. 2016), the wake of the single turbine shows a faster recovery in the convective case, and a slower one in the stable case, compared to the neutral one. The same trend holds for the cumulative wake of the two turbines. As explained by Abkar and Porté-Agel (2015), the relatively faster wake recovery in the convective case can be related to enhanced flow mixing due to higher turbulent intensity and fluxes, and positive buoyancy in the incoming boundary layer flow. Similarly, negative buoyancy and smaller turbulent fluxes in the stable boundary layer lead to the slower wake recovery in the stable case compared to the neutral case. The difference between the convective and neutral cases is higher than that between the stable and neutral ones. This is consistent with the stability characteristics discussed in the previous section, which showed strong convective conditions, but weak stable conditions in the measurement region of the test section for the prescribed inlet temperature profiles.

**Fig. 7:**
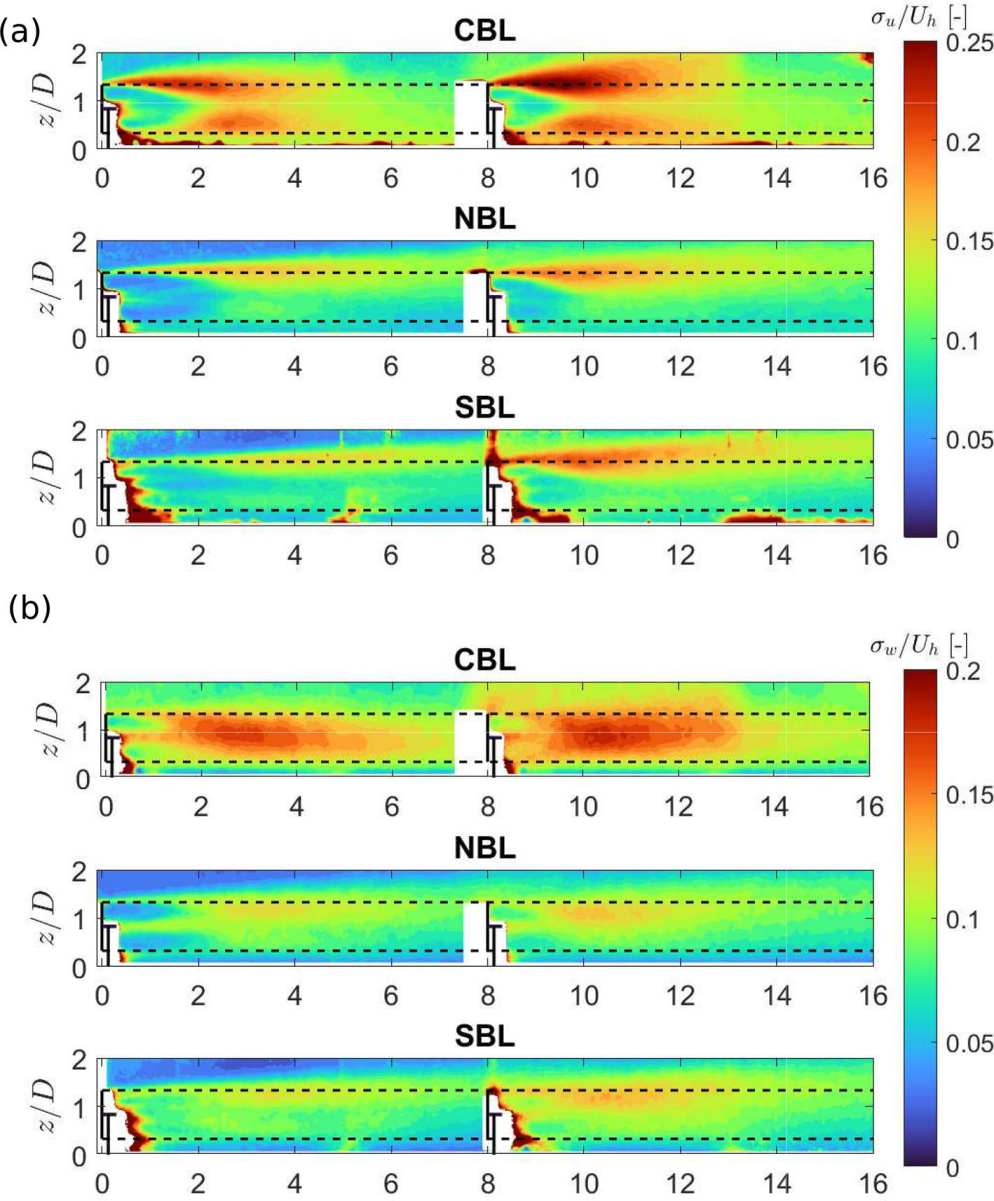
Contours of the streamwise (a) and vertical (b) turbulence intensity in the turbine wakes for different thermal stability conditions.

It is well understood that thermal stability can also affect turbulence intensity in the turbine wake (Porté-Agel et al. 2020). Figure 7 (a) shows the contours of the streamwise turbulence intensity in the turbine wakes obtained from the experiments. The streamwise turbulence intensity is observed to be strongly dependent on thermal stability, which is interesting, as in the boundary layer flow, the streamwise turbulence intensity was observed to be similar between different stability conditions (see Fig. 3(d)). It shows that even if the incoming streamwise turbulence intensity is the same under different thermal conditions, its magnitude and spatial distribution in the turbine wake are still affected by thermal stability. This further points out to the fact that the turbine-added turbulence intensity is affected by thermal stability. For the NBL case, a peak around the rotor top tip height can be observed, which increases in magnitude in the cumulative wake of two turbines. For the SBL case, a similar distribution of streamwise turbulence intensity is observed. The magnitude of streamwise turbulence intensity behind the first turbine is slightly lower in the SBL case than in the NBL one, which can be related to the weakly stable boundary layer conditions. In the cumulative wake of two turbines, on the other hand, the streamwise turbulence intensity is slightly higher in the SBL case than in the NBL one. As explained by Chamorro and Porté-Agel (2010), in the wake the shear production of turbulence can dominate over the turbulence suppression due to negative buoyancy due to higher velocity deficit in the stable case, leading to higher turbulence in the wake compared to the neutral case. In the CBL case, the streamwise turbulence intensity shows the highest magnitude compared to the NBL and SBL cases. In addition, the turbulence intensity distribution shows two peaks, one at the turbine top tip level and the other at the bottom tip level. This is consistent with relatively higher shear around both tip levels of the rotor in the CBL case, compared to the NBL and SBL cases, which show smaller shear around the bottom tip level (leading to one pronounced peak in turbulence intensity around the top tip level). In addition, in the far wake, the turbulence intensity distribution is observed to flatten, which is consistent with the faster wake recovery in that case. In addition to the streamwise turbulence intensity, the contours of vertical turbulence intensity are shown in Fig. 7 (b). Consistent with previous studies (Zhang et al. 2012; Xie and Archer 2015), the magnitude of vertical turbulence intensity is found to be smaller than that of streamwise turbulence intensity in respective cases. Comparing different cases, the magnitude of vertical turbulence intensity shows similar trends to the streamwise turbulence intensity, with the highest magnitude in the CBL case. While the NBL and SBL cases show a peak value around the rotor top tip, for the CBL case a peak around the hub height of the turbine is observed. This is consistent with the results of Hancock et al. (2014), and can be related to the difference in the mean flow shear in vertical velocity.

### Two-point Correlations

Spatial correlations in turbulent flows can give an estimate of the characteristic size of turbulent length scales. For wind energy, these correlations can have an impact on the unsteady loads experienced by turbines (Dörenkämper et al. 2015; Tian et al. 2018). Bastankhah and Porté-Agel (2017c) investigated the two-point streamwise velocity fluctuation correlation in a turbine wake at the hub level in neutral conditions. They found that the spatial coherence is smaller in the near-wake and increases in magnitude in the far wake. In addition, they showed that these spatial correlations are affected by the meandering of the wake. More recently, Placidi et al. (2023) investigated the effect of stable thermal conditions on two-point correlations of streamwise velocity fluctuations using laser-Doppler velocimetry in the wake of single and two fully-aligned wind turbines. They showed that, around the rotor top tip level, stratification had a strong effect on the two-point correlations. Here, we investigate the effect of three different thermal stability conditions on two-point correlations of the streamwise and vertical velocity fluctuations in the wake of a single turbine and the cumulative wake of two fully-aligned turbines. The two-point correlations are defined as:

4 $$\begin{aligned} R_{uu} = \frac{\overline{u'(x_{ref},z_{ref})u'(x,z)}}{\sqrt{\overline{u'(x_{ref},z_{ref})^2} \times \overline{u'(x,z)^2}}}, \end{aligned}$$

5 $$\begin{aligned} R_{ww} = \frac{\overline{w'(x_{ref},z_{ref})w'(x,z)}}{\sqrt{\overline{w'(x_{ref},z_{ref})^2} \times \overline{w'(x,z)^2}}}, \end{aligned}$$

where $R_{uu}$ and $R_{ww}$ are the two-point correlations of the streamwise and vertical velocity fluctuations, $u'$ and $w'$ represent the instantaneous velocity fluctuations in the streamwise and vertical velocity components, and $(x_{ref},z_{ref})$ are the coordinates for the reference point chosen for the correlation. In this study, the reference points are chosen in the near and far wake of the single and cumulative wakes at the turbine top tip, hub, and bottom tip heights.

**Fig. 8:**
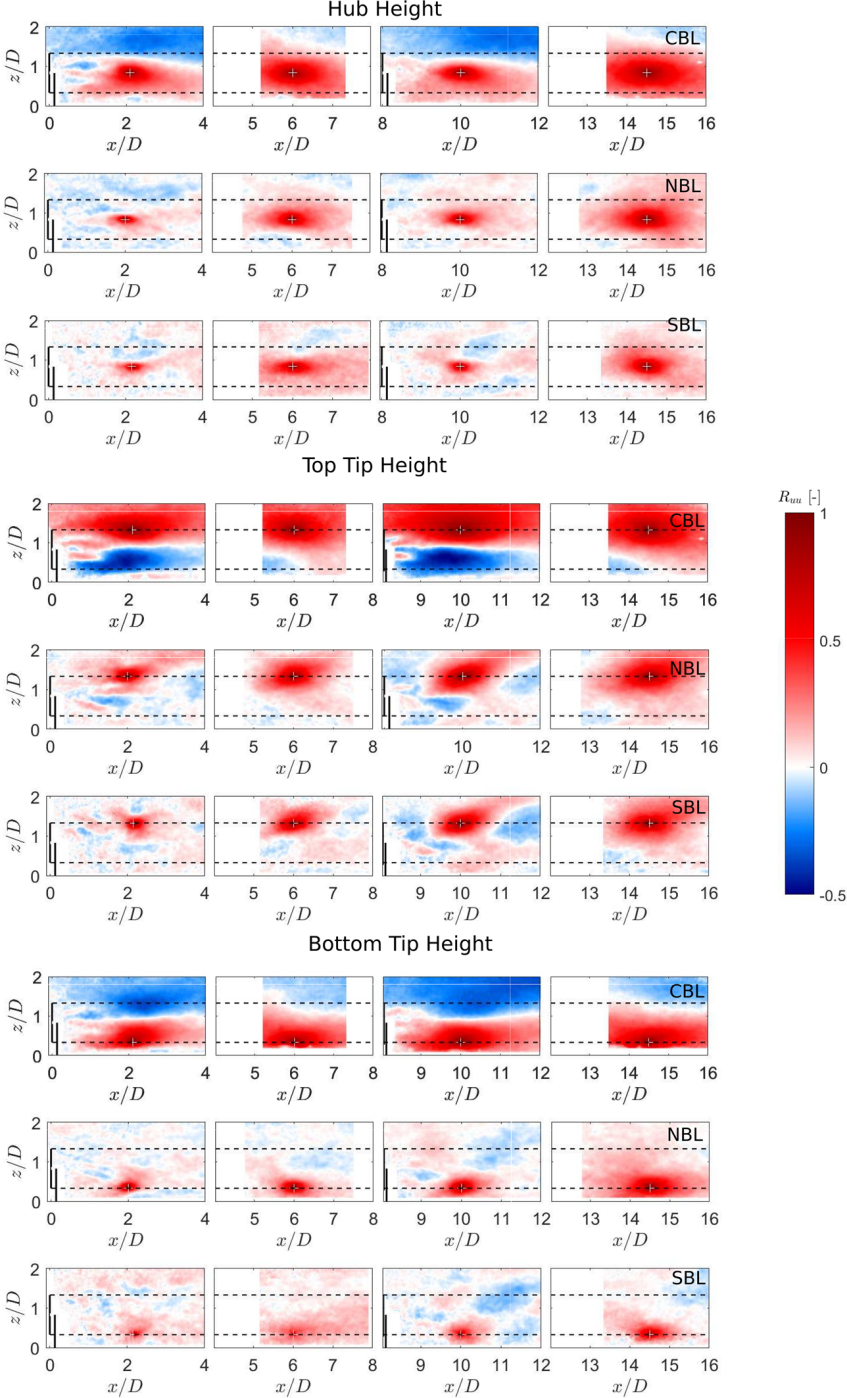
Contours of the two-point correlations of streamwise velocity fluctuations in the wake. White plus signs show the reference location.

**Fig. 9:**
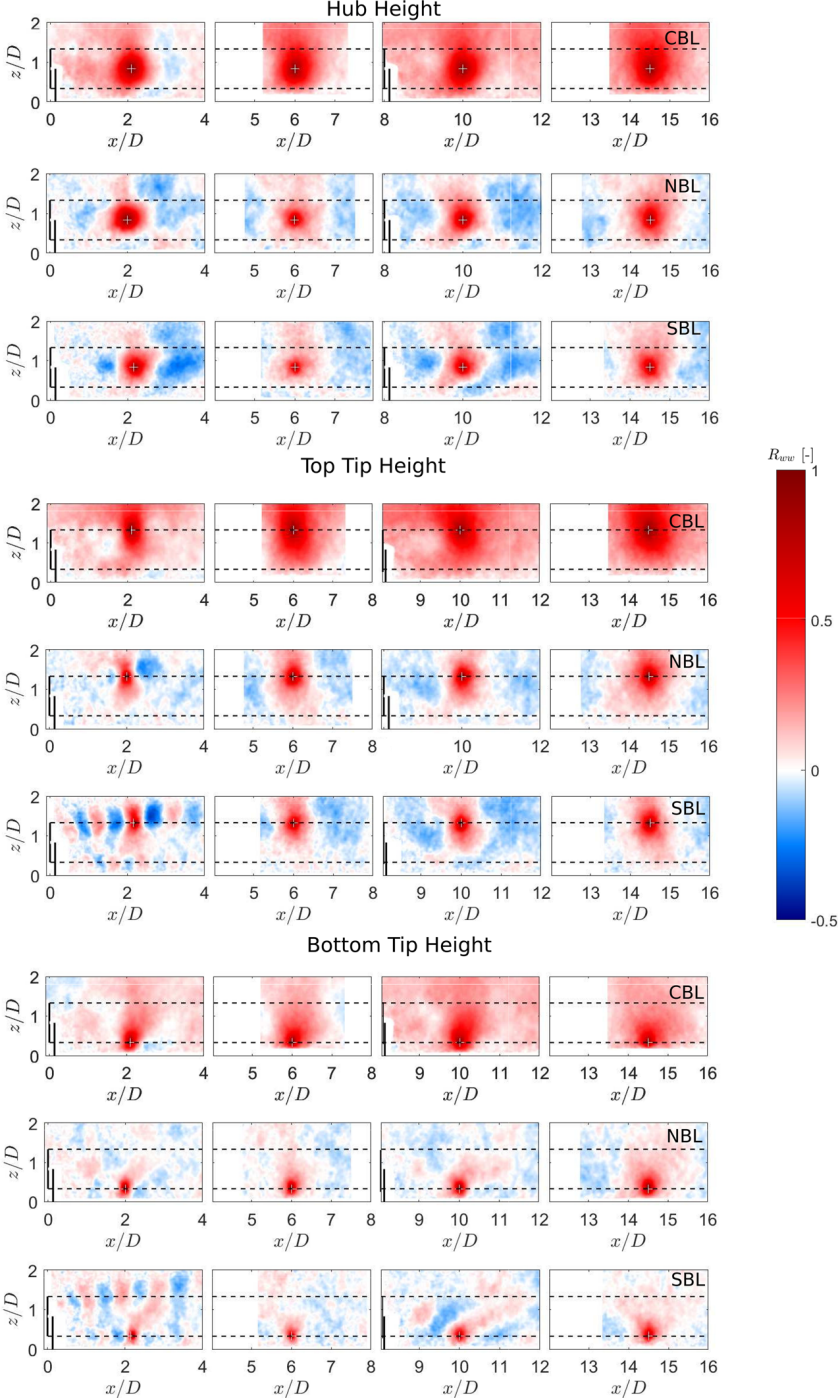
Contours of the two-point correlations of vertical velocity fluctuations in the wake. White plus signs show the reference location.

Figure 8 shows the contours of the two-point correlations in streamwise velocity fluctuations with different reference positions. Comparing different thermal stability conditions, it can be observed that the spatial extent of correlated regions is highest in the convective case, whereas the stable case shows the lowest correlation regions, pointing towards largest turbulent structures in convective case and smallest in stable one. In addition, this indicates that even for the same streamwise turbulence intensity in all the boundary layers (as shown in Fig 3(d)), the streamwise turbulent correlations in the wake are affected by the thermal stability. The two-point correlation in the streamwise velocity fluctuations has a smaller extent in the near wake and a larger one in the far wake. In addition, the correlated region is larger in the cumulative wake than in the single turbine wake. Consistent with previous studies (Bastankhah and Porté-Agel 2017a; Dar and Porte-Agel 2020), the shear layers developing around the hub and rotor tips result in smaller correlated region in the near wake than in the far wake. In addition, as the turbulent mixing is higher in the cumulative wake of two turbines, the resulting correlation is stronger, which indicates an increase in the turbulence length scales. The correlated region is also found to have a larger extent when the reference point is chosen at the rotor top tip height, and a smaller extent around the rotor bottom tip. As discussed by Placidi et al. (2023), this is likely due to the increase in turbulent length scales with height in the incoming boundary layer flow. In terms of the structure of the correlated regions, it is observed to be very similar in the neutral and stable cases, with the only difference being the size of the correlated region. For the convective case, on the other hand, in addition to the increase in the size of the correlated region compared to neutral and stable cases, an anti-correlated region can also be observed. This anti-correlated region exists for all reference point locations. Bastankhah and Porté-Agel (2017c) made a similar observation for the two-point correlations in a spanwise plane at the hub height for a turbine wake under neutral conditions. They related this to the meandering of the wake. For the vertical plane, the meandering is usually small under neutral conditions due to boundary layer shear. However, under convective conditions, the vertical boundary layer profile is relatively uniform due to high turbulent mixing. This indicates that the anti-correlated regions observed for the convective case could be related to the enhanced wake meandering.

Figure 9 shows the contours of the two-point correlations in the vertical velocity fluctuations for different thermal conditions. Similar to the streamwise velocity correlations, the vertical velocity correlations are found to be dependent on thermal stability, with the largest correlated region in the convective case and the smallest in the stable one. The vertical two-point correlation shows a stronger dependency on the vertical position of the reference point than the streamwise correlation. The convective case shows positive correlation throughout each of the field of views, indicating turbulent length scales on the order of several rotor diameters in the turbine wake. For the neutral case, the vertical correlation extends to approximately one rotor diameter in the streamwise directions for the reference point at the hub or rotor top tip height. At the bottom tip height, the correlation region is very small, indicating smaller vertical length scales in this region. The positive correlation region is surrounded by a negative one on both sides for reference heights at the turbine hub and top tip level. It could be related to the periodic vortices shed from the turbine blades. For the stable case, a very strong signature of the tip vortices can be seen in the correlation when the reference point is chosen in the near wake at the top tip height of the first turbine. This is consistent with the fact that the tip vortices are more stable and last longer in this case. A similar pattern, although weaker in magnitude, can also be seen when the reference point is chosen at the bottom tip height. This indicates that the two regions are part of the same structure - the helical vortices shed from the turbine blades.

### Far-wake Modeling

Analytical modeling of the streamwise wake velocity deficit is important for wind farm layout optimization due to low computational cost and reasonable accuracy. In this section, we test the standard Gaussian model (Bastankhah and Porté-Agel 2014, 2016) to predict the streamwise wake velocity deficit behind one and two turbines. The approach tested here is different from that of Abkar and Porté-Agel (2015), who used the wake width from large eddy simulation as an input for the analytical model. Instead, here we attempt to model it using the empirical relation obtained by Brugger et al. (2019).

According to the Gaussian model, the streamwise wake velocity deficit has the following form:

6 $$\begin{aligned} \frac{U_{in}-U_w}{U_{in}} = C(x)e^{-(\frac{r^2}{2\sigma (x)^2})}, \end{aligned}$$

where *C*(*x*) is the normalized maximum velocity deficit, *r* is the radial distance from the wake center and $\sigma$ is the wake width. The normalized maximum velocity deficit is computed from the expression derived by Bastankhah and Porté-Agel (2014):

7 $$\begin{aligned} C(x) = 1-\sqrt{1-\frac{C_T}{8(\sigma (x)/D)^2}}, \end{aligned}$$

where $C_T$ is the turbine thrust coefficient. In the current experiments, the thrust coefficient of the turbine is measured under low Reynolds number in neutral conditions using a torque sensor mounted at the bottom of the turbine tower. The thrust coefficient at the optimum tip speed ratio is 0.725, which is lower than the 0.82 value obtained for the same turbine at a higher Reynolds number (Bastankhah and Porté-Agel 2016). The wake width $\sigma$ is obtained using the linear relation:

8 $$\begin{aligned} \frac{\sigma (x)}{D} = k\frac{x-x_{nw}}{D} + \frac{\sigma _{nw}}{D}, \end{aligned}$$

where *k* is the wake growth rate, $x_{nw}$ is the near wake length and $\sigma _{nw}$ is the wake width at the end of the near wake. The wake growth rate *k* is often related to the streamwise turbulence intensity in the incoming boundary layer flow (Niayifar and Porté-Agel 2016; Brugger et al. 2019). Here, we use the relation suggested by Brugger et al. (2019), which states:

9 $$\begin{aligned} k=0.3I_u, \end{aligned}$$

where $I_u$ is the streamwise turbulence intensity at the hub height of the turbine in the incoming boundary layer. According to Bastankhah and Porté-Agel (2016), the wake width at the end of the near wake is $1/\sqrt{8}$, whereas the near wake length can be obtained as:

10 $$\begin{aligned} \frac{x_{nw}}{D} = \frac{1+\sqrt{1-C_T}}{\sqrt{2}(4\alpha I_u+2\beta (1-\sqrt{1-C_T}))}, \end{aligned}$$

where $\alpha$ and $\beta$ are model constants given as 0.58 and 0.077, respectively. For the waked turbine, the added streamwise turbulence intensity of the upstream turbine is accounted for by using the Frandsen model (Frandsen 2007):

11 $$\begin{aligned} I_{a} = \frac{1}{a_1+\frac{a_2}{\sqrt{C_T}}\frac{x}{D}}, \end{aligned}$$

where $a_1 = 1.5$ and $a_2 =0.8$. The wake growth rate is then computed as: $k = 0.3\sqrt{I_u^2+I_a^2}$. Similarly, the impact of added turbulence of the wake of the upstream turbine is accounted for in the estimation of the near wake length of the second turbine. Finally, to compute the cumulative wake velocity deficit, the linear superposition method of Niayifar and Porté-Agel (2016) is used.

**Fig. 10:**
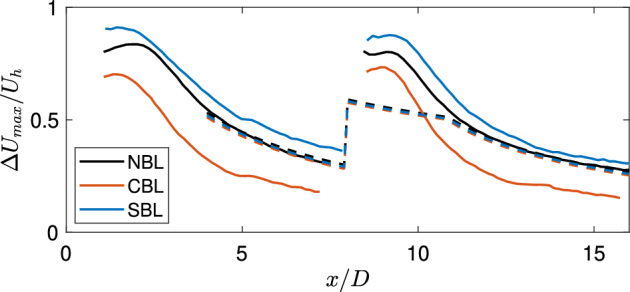
Comparison of the normalized maximum streamwise velocity deficit between the experiments (solid lines) and standard Gaussian model (dashed lines) in the turbine wake for different thermal stability cases.

Fig 10 compares the normalized maximum streamwise velocity deficit between the experiments and the analytical model. As can be seen, the model can predict the maximum normalized wake velocity deficit with good accuracy for the neutral case. However, for the stable and convective cases, the model cannot capture the wake deficit. This can be related to the fact that the model relies on the streamwise turbulence intensity as a surrogate for thermal stability. In these experiments, the streamwise turbulence intensity across the rotor is comparable between different cases, leading to similar predictions from the analytical model for all the cases. This emphasizes on the fact that thermal stability can affect the wake deficit independently from the streamwise turbulence intensity. Therefore, using the streamwise turbulence intensity as a surrogate for thermal stability can lead to significant errors in the estimation of wake losses. In the cumulative wake of two turbines, the wake deficit is predicted reasonably well for the stable case, which can be related to the relatively small difference between the neutral and stable cases.

Some recent studies have shown that the wake width or expansion rate in the lateral and vertical directions is related to the turbulence intensity in the respective directions (see e.g., Vahidi and Porté-Agel (2022)). In the current study, we observed a clear change in the vertical turbulence intensity with thermal stability - with values decreasing for SBL, and increasing for CBL compared to the NBL case. Although not captured, a similar behavior would be expected of the lateral turbulence intensity. Therefore, we attempt to improve the analytical framework by relating the wake expansion rate *k*, near wake length $x_{nw}$ and added turbulence intensity with the vertical turbulence intensity instead of the streamwise one. For this purpose, we use the ratio of streamwise to vertical turbulence intensity in the neutral boundary layer from experiments to translate the relation for wake growth rate and near wake length in terms of vertical turbulence intensity, as these relations are initially obtained under neutral conditions. From the neutral boundary layer data, we obtain the following ratio between streamwise and vertical turbulence intensity at the hub height:

12 $$\begin{aligned} \gamma = \frac{I_{u}}{I_{w}} = 1.65. \end{aligned}$$

Plugging equation 12 in equations 9 and 10 yields the updated relations for wake growth rate and near wake length in terms of vertical turbulence intensity:

13 $$\begin{aligned} k = 0.495 I_w, \end{aligned}$$

and

14 $$\begin{aligned} \frac{x_{nw}}{D} = \frac{1+\sqrt{1-C_T}}{\sqrt{2}(4\alpha ' I_w+2\beta (1-\sqrt{1-C_T}))}, \end{aligned}$$

where $\alpha '=\alpha \gamma \approx 0.96$. The added streamwise turbulence model was originally obtained by fitting equation 11 to experimental data to obtain the model coefficients $a_1$ and $a_2$ (Frandsen 2007). Here, we keep $a_2$ as in the original model, but calibrate $a_1$ on the added vertical turbulence intensity in the wake of the first turbine under neutral conditions. This results in $a_1 = 4.5$. Once the updated relations for the wake growth rate, near wake length, and added turbulence are obtained, they are tested for all cases to test their ability to relate wake deficit with the vertical turbulence intensity and validity under different stability conditions. This approach uses the vertical turbulence intensity as a surrogate for thermal stability rather than the usual approach of using streamwise turbulence intensity (Krutova et al. 2020).

**Fig. 11:**
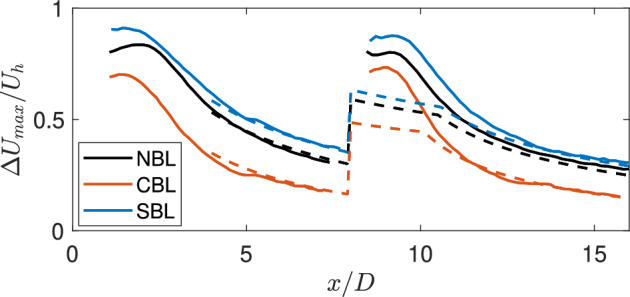
Comparison of the normalized maximum streamwise velocity deficit between the experiments (solid lines) and modified Gaussian model (dashed lines) in the turbine wake for different thermal stability cases.

**Fig. 12:**
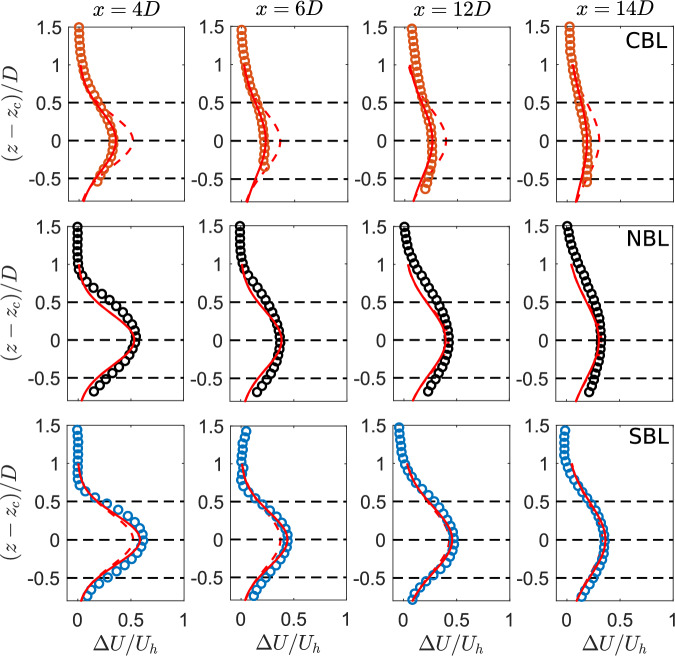
Comparison of the vertical profiles of the normalized averaged streamwise velocity deficit between the experiments (circles), standard (dashed lines) and modified (solid lines) Gaussian model. The horizontal dashed lines show the rotor tip, hub and bottom heights.

Fig 11 compares the normalized maximum streamwise velocity deficit between the experiments and the modified Gaussian model for different stability cases. It can be immediately identified that the new approach can significantly improve the prediction of the normalized maximum velocity deficit for all cases compared to the standard approach. It can also capture the faster and slower wake recovery in the convective and stable cases, respectively, compared to the neutral one. In addition, the cumulative wake deficit maximum is also predicted with good accuracy in the far wake. Finally, Fig 12 compares the vertical profiles of the normalized averaged streamwise velocity deficit between the experiments, and the standard and modified Gaussian modeling frameworks. For the neutral case, both methods yield similar results, showing that they are interchangeable for that case. For the convective case, the modified framework can correctly predict the maximum deficit and wake width compared to the standard one (which overpredicts the maximum deficit). Similarly, for the stable case, there is also an improvement in the prediction of the velocity deficit profile using the new approach. Therefore, the proposed analytical framework can predict the wake velocity deficit behind the single and cumulative wake velocity deficit under different thermal stability conditions.

## Concluding Remarks

In this work, we present first results from a newly commissioned thermal stratification system at the boundary layer wind tunnel of EPFL. The thermal stability is achieved by heating or cooling of the floor combined with the heating and cooling of the air at 16 equidistant heights at the start of the convergent section of the wind tunnel test section. The current experiments are performed 20 *m* downstream of the test section inlet where the turbulent boundary layer is fully developed. Three different thermal stability conditions are set up corresponding to neutral, strongly convective, and weakly stable conditions. The wake of two fully aligned turbines with an inter-turbine spacing of 8 rotor diameters is investigated.

Results show faster and slower recovery of the wake deficit in the single and cumulative wake conditions for the convective and stable conditions compared to the neutral one, respectively. In addition, an increase and slight decrease in the streamwise turbulence intensity in the turbine wake is observed for the convective and stable cases compared to the neutral one, respectively. The turbulent length scales are investigated using two-point correlations of streamwise and vertical velocity fluctuations in the turbine wake under different thermal conditions. The correlation in the streamwise velocity fluctuations increases in the convective condition compared to the neutral one, and displays strong anti-correlated regions, which indicate enhanced meandering in the vertical direction under convective conditions. The stable case shows similar trends to the neutral one, but a smaller correlated region. For correlation in the vertical velocity fluctuations, the convective case shows high positive correlation throughout the streamwise extent of the measurement plane. For the stable case, on the other hand, a strong signature of tip vortices can be identified behind the first turbine. The correlation further downstream is shown to be weaker than for the neutral case.

The experimental data is used to test the Gaussian analytical wake model. It is shown that in its standard formulation, the Gaussian model cannot predict the wake velocity deficit for the stable and convective conditions. This is related to the fact that, in the current experiments, the streamwise turbulence intensity in the boundary layer is the same for all the cases. As the streamwise turbulence intensity is used to implicitly account for thermal stability effects in the Gaussian model, it fails to capture these effects. To resolve this, the neutral boundary layer data is used to re-formulate the relations for wake expansion, near wake length, and wake-added turbulence in terms of the vertical turbulence intensity, which shows clear dependency on stability. The re-formulated relations are then used to predict the velocity deficit for all cases. It is, thereby, observed that the model with the re-formulated relations is able to correctly predict the wake velocity deficit of the single and cumulative wake with good accuracy.

## Data Availability

The experimental data can be made available by contacting the corresponding author upon reasonable request.
